# Correlation between Anterior Mitral Annular Plane Systolic Excursion and Left Atrial Appendage Stasis in Patients with Nonvalvular Atrial Fibrillation

**DOI:** 10.31083/j.rcm2507236

**Published:** 2024-06-28

**Authors:** Jia-Li Fan, Hai-Peng Wang, Yao Lu, Heng Wang, Chang-Sheng Ma

**Affiliations:** ^1^Department of Echocardiography Cardiology, The First Affiliated Hospital of Soochow University, 215031 Suzhou, Jiangsu, China; ^2^Department of Cardiology, The First Affiliated Hospital of Soochow University, 215031 Suzhou, Jiangsu, China; ^3^Department of Medicine, Jiangsu University, 212013 Zhenjiang, Jiangsu, China

**Keywords:** transesophageal echocardiography, atrial fibrillation, left atrial appendage, spontaneous echocardiographic contrast, left atrial appendage function

## Abstract

**Background:**

Atrial fibrillation (AF) can lead to a decline in left atrial appendage (LAA) function, potentially increasing the likelihood of LAA thrombus (LAAT) and spontaneous echo contrast (SEC). Measuring LAA flow velocity through transesophageal echocardiography (TEE) is currently the primary method for evaluating LAA function. This study aims to explore the potential correlation between anterior mitral annular plane systolic excursion (aMAPSE) and LAA stasis in patients with non-valvular atrial fibrillation (NVAF).

**Methods:**

A total of 465 patients with NVAF were enrolled between October 2018 and November 2021. Transthoracic echocardiography (TTE) and TEE were performed before scheduled electrical cardioversion. Propensity score matching (PSM) was used to balance confounders between the groups with and without LAAT/dense SEC.

**Results:**

Patients in the LAAT/dense SEC group showed increased left atrial (LA) diameter, LAA area, alongside reduced left ventricular ejection fraction (LVEF), LAA velocity, conjunction thickening ratio, aMAPSE, and LAA fraction area change (FAC) compared to those in the non-LAAT/dense SEC group. Multivariate logistic regression analysis identified aMAPSE and LAA FAC as independent predictors for LAAT/dense SEC. Specifically, an aMAPSE of <6.76 mm and an LAA FAC of <29.65% predicted LAAT/dense SEC with high diagnostic accuracy, demonstrated by an area under the curve (AUC) of 0.81 (sensitivity 0.81, specificity 0.80) for aMAPSE, and an AUC of 0.80 (sensitivity 0.70, specificity 0.84) for LAA FAC.

**Conclusions:**

Both aMAPSE and LAA FAC independently correlated with and accurately predict LAAT/dense SEC. Incorporating aMAPSE into routine TEE evaluations for LAA function alongside LAA flow velocity is recommended.

## 1. Introduction

Non-valvular atrial fibrillation (NVAF) significantly increases the risk of stroke or systemic embolism [1]. In NVAF patients, more than 90% of thrombi originate within the left atrial (LA) appendage (LAA) [2]. Both LAA thrombus (LAAT) and LAA spontaneous echocardiographic contrast (SEC) are correlated with an increased risk of thrombus formation and thromboembolic events [3, 4, 5, 6]. Presently, two-dimensional (2D) transesophageal echocardiography (TEE) is the principal method for diagnosing and excluding the presence of LAAT and LAA SEC [7].

As atrial fibrillation (AF) progresses, LAA function tends to deteriorate, heightening the risk of LAAT and LAA SEC [8]. Notably, LAA flow velocity serves as an indirect measurement for evaluating LAA function and is widely utilized in clinical practice. Specifically, an LAA emptying velocity (LAA-EV) of 40 cm/s or less is associated with an increased risk of thrombus formation within the LAA, potentially leading to embolic events [9]. Moreover, velocities of 20 cm/s or below frequently indicate the presence of SEC in the LA/LAA [5, 10, 11, 12]. Besides LAA flow velocity, the LAA emptying fraction (LAAEF) measured by TEE has been shown to correlate with LAAT in NVAF patients with a low CHADS2 score [13].

**Fig. 1.**
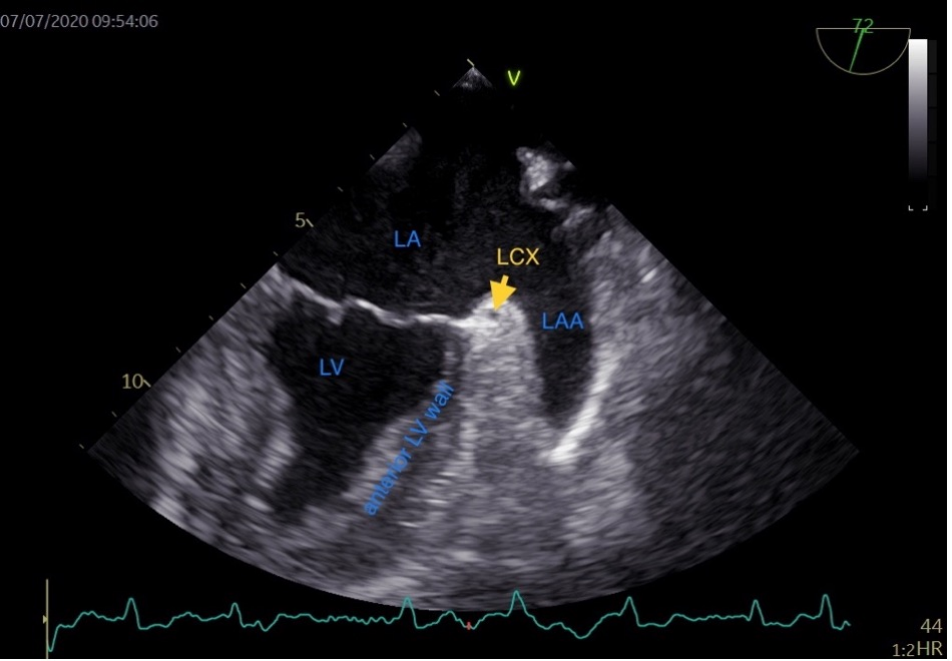
**Transesophageal echocardiographic view of the left atrial appendage.** LA, left atrium; LV, left ventricle; LCX, left circumflex artery; LAA, left atrial appendage; HR, heart rate.

Typically, the LAA extends between the anterior and lateral walls of the LA, with its tip directed anterosuperiorly. This orientation causes it to overlay the left side of the right ventricular outflow tract, the pulmonary trunk, or to be in proximity to the main branch of the left coronary or the left circumflex artery (LCX). The LAA’s somewhat flattened structure results in its underside often covering the anterior and lateral walls of the left ventricle, while its topside is positioned under the fibrous pericardium [14]. In the TEE view of the LAA, the base of the left ventricle (LV) anterior wall is adjacent to the LAA (Fig. 1). The LAA compresses during LV diastole and stretches during LV systole. We speculate that the structure and function of the base of the LV anterior wall may influence LAA function. Consequently, we hypothesize that the anterior mitral annular plane systolic excursion (aMAPSE) can be utilized to assess LAA function. Additionally, the conjunction of LA and LAA is composed of LAA wall and fibrous pericardium, which vary in thickness with the systole and diastole phases of the LAA. We speculate that the LAA conjunction thickness ratio may reflect LAA function and correlate with LAA stasis. Given that the base of the LV wall compresses and stretches the LAA, we included the base of LV wall in measuring the LAA conjunction thickness.

The aim of this study was to investigate the correlation between LAA functional parameters and the presence of LAAT or dense SEC in patients with NVAF. Further, we compare the diagnostic value of aMAPSE with standard LAA functional parameters for detecting LAAT and dense SEC.

## 2. Methods

### 2.1 Study Population

The study was approved by the institutional enrolling board of the first affiliated hospital of Soochow university. Between October 2018 and November 2021, we prospectively studied 465 patients with NVAF after providing informed written and verbal consent, 2D-TEE examination was performed for them to exclude the presence of LAA SEC or thrombus before radiofrequency ablation. This study excluded patients who met any of the following criteria: moderate to severe valvular disease, hypertrophic cardiomyopathy, unsuccessful TEE procedures, and instances with images that did not meet quality standards. Collected clinical data encompassed demographic information, current medication regimens, and previous medical history. The ${\mathrm{CHA}}_{2}$${\mathrm{DS}}_{2}$-VASc score was calculated for each patient [15].

### 2.2 Transthoracic Echocardiography

We performed TTE using the General Electric Vivid E95 device (GE Vingmed Ultrasound AS, Horten, Norway) and a sector array M5Sc (2.5–3.5 MHz) transducer (GE Vingmed Ultrasound AS, Horten, Norway). Images were acquired to measure left atrial diameter, left ventricular end-diastolic diameter (LVEDd), left ventricular ejection fraction (LVEF), E/e’ and systolic pulmonary arterial pressure (sPAP). The LA diameter was measured in the parasternal long-axis view at the ventricular end-systole. The LVEF was measured using Simpson’s method, which was used as a standard index of global LV systolic function. In addition, E/e’ was the ratio of early diastolic mitral inflow to mitral annular tissue velocities (the average of septal e’ and lateral e’). All echocardiographic measurements for AF patients used in the analysis were averaged from 3 heart beats.

### 2.3 Transesophageal Echocardiography

**Fig. 2.**
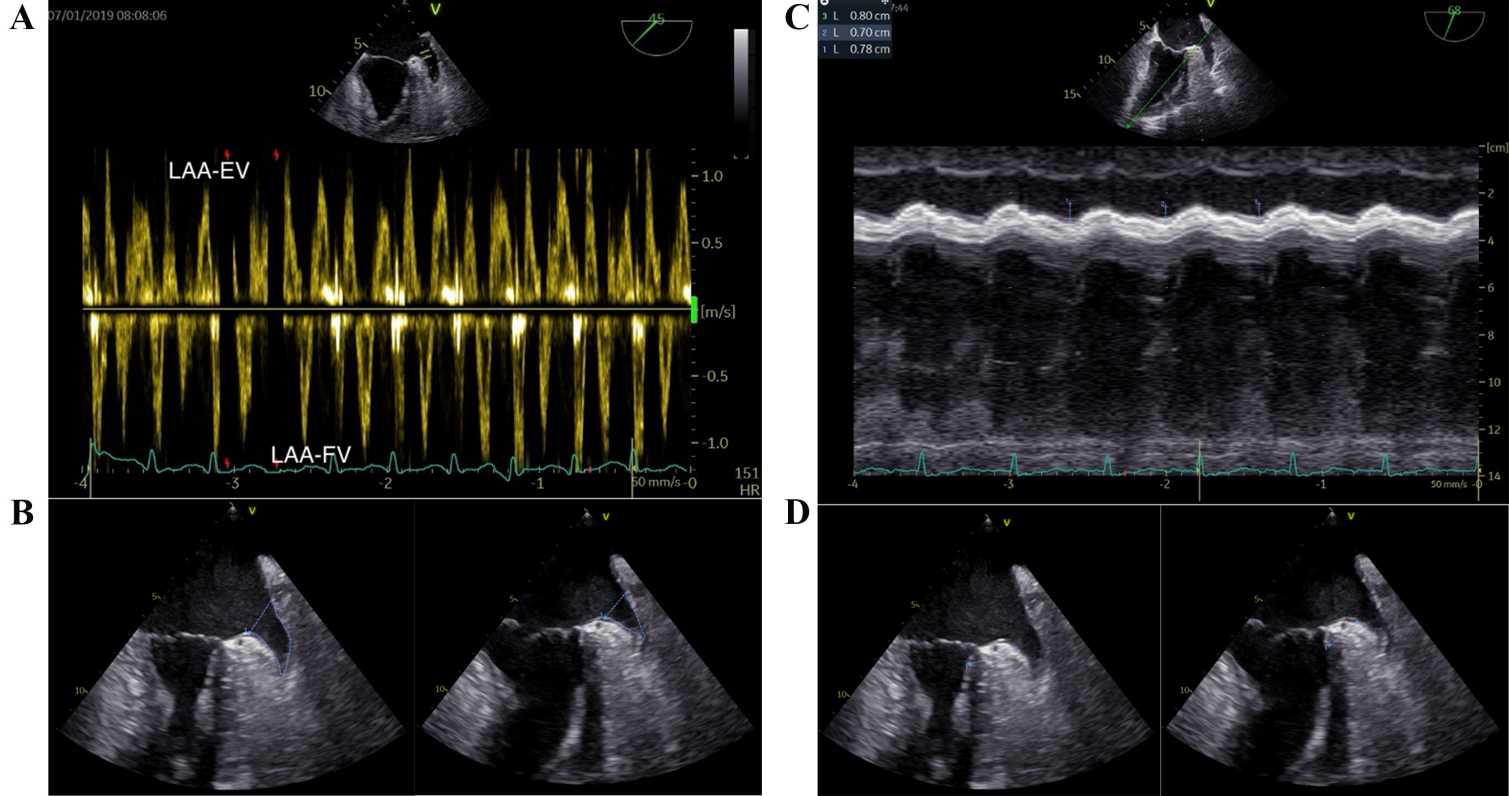
**Key transesophageal echocardiographic parameters in the LAA function assessment.** (A) LAA-EV and LAA-FV. (B) LAA fraction area change (maximum LAA area and minimum LAA area). (C) aMAPSE. (D) LAA conjunction thickening ratio (maximum LAA conjunction thickness and minimum LAA conjunction thickness). LAA, left atrial appendage; LAA-EV, left atrial appendage emptying velocity; LAA-FV, left atrial appendage filling velocity; aMAPSE, anterior mitral annular plane systolic excursion; HR, heart rate.

The TEE procedures were performed consistently by a single operator, utilizing a GE Vivid E95 apparatus (GE Vingmed Ultrasound AS, Horten, Norway) equipped with a multiplane 6VT (3.0-8.0 MHz) transducer. Optimal visualization of the LAA was achieved through the mid-esophageal approach, angling the probe (usually between 45° and 90°) to maximize the length from apex to orifice. The imaging frame rate was maintained between 60 and 90 frames per second. Typically, five cardiac cycles were analyzed for both 2D imaging and pulsed wave (PW) Doppler studies. The PW-Doppler was employed to measure LAA flow at the orifice, with appropriate adjustments made to gain and filter settings. Both LAA-EV and LAA filling velocity (LAA-FV) were recorded (Fig. 2A). The LAA fractional area change (LAA FAC) was determined using the formula: (maximum LAA area – minimum LAA area)/maximum LAA area (Fig. 2B). The measurement of the LAA ostium was taken from the circumflex artery to a superior point situated 1 to 2 cm within the left lateral ridge, while the depth of the LAA is gauged from this line to the LAA’s apex [16]. The aMAPSE was measured between end-diastole and peak systole using M-mode in the direction of anterior left ventricle longitudinal extension, which was assessed from the apical approach (Fig. 2C). Conjunction thickness was measured from 0–3 mm beneath LCX and perpendicular to anterior wall of left ventricle. The conjunction thickening ratio was defined as conjunction thickening gradient divided by maximum thickness of LAA conjunction (Fig. 2D). In cases of AF, the mean values from three cardiac cycles were evaluated.

SEC was identified by the presence of dynamic, swirling, smoke-like echoes in the LAA cavity [17]. The severity of SEC was classified into five grades using predefined criteria: none (0), mild (1+), moderate (2+), moderate to severe (3+), and severe (4+) [18]. LAAT was defined from various imaging angles as a clearly delineated, solid echo density that stood out acoustically from the surrounding endocardium and pectinate muscles, exhibiting less heterogeneity and dynamism compared to sludge [19, 20].

### 2.4 Statistical Analysis

Analyses were performed using the STATA 17.0 (StataCorp LP, College Station, TX, USA). In the current study, patients were categorized into two groups: those with LAAT/dense SEC and those without (non-LAAT/dense SEC). Propensity score matching (PSM) was used to adjust for confounders between the two groups. The variables included for matching were age, gender, body surface area (BSA), persistent AF, prior embolic events including transient ischemic attack (TIA) or ischemic stroke, hypertension, diabetes, congestive heart failure, vascular disease, and use of anticoagulants. We matched the two groups in a 1:1 ratio with a caliper width of 0.2. Normally distributed continuous variables were presented as mean ± SD, while those not normally distributed were presented as median with inter-quartile ranges (IQR, 25th–75th percentile). Categorical variables were expressed as numbers and percentages. For comparison between groups, continuous variables were compared using the *T*-test or Mann–Whitney U test as appropriate. Categorical variables were analyzed using the Chi-square test. A difference was considered significant when *p* < 0.05. We applied univariable and multivariable logistic regression models to identify the predictors for LAAT/dense SEC in the matched cohort. To avoid data overfitting and collinearity, variables with *p* < 0.05 and variance inflation factor (VIF) <5 in the univariate models were included in the multivariate model. The optimal cutoff values for assessing the sensitivity and specificity of echocardiographic indicators in identifying LAAT/dense SEC were obtained from receiver operating characteristic (ROC) curve analysis. The correlation between aMAPSE and clinical and echocardiographic parameters was analyzed by univariable and multivariable linear regression models in the overall population.

## 3. Results

### 3.1 Characteristics of the Population

Among the 465 study subjects, LAAT/dense SEC was identified by TEE in 107 (23.01%). Before propensity score matching, we detected differences in several baseline variables between the two groups. After applying propensity score matching, 56 patients were selected for each of the LAAT/dense SEC and non-LAAT/dense SEC groups. Post-matching analysis revealed no significant differences in demographic and clinical characteristics between the groups (Table 1).

**Table 1.** **Comparative demographic and clinical characteristics of LAAT/Dense SEC and Non-LAAT/Dense SEC groups following propensity score matching**.

Variables		Before matching			After matching		
		Non-LAAT/dense SEC (n = 358)	LAAT/dense SEC (n = 107)	*p*	Non-LAAT/dense SEC (n = 56)	LAAT/dense SEC (n = 56)	*p*
Age (y)		65.00 (58.00, 69.00)	67.00 (63.00, 72.00)	<0.001	67.00 (62.00, 72.00)	67.50 (62.00, 71.00)	0.760
Gender				0.007			0.570
	Female	135 (37.71%)	56 (52.34%)		28 (50.00%)	25 (44.64%)	
	Male	223 (62.29%)	51 (47.66%)		28 (50.00%)	31 (55.36%)	
BSA ($m^{2}$)		1.87 (0.18)	1.82 (0.19)	0.006	1.84 (0.17)	1.84 (0.19)	0.930
Persistent AF		156 (43.58%)	99 (92.52%)	<0.001	50 (89.29%)	50 (89.29%)	1.000
Previous embolic events, TIA, or ischemic stroke		32 (9.04%)	10 (9.43%)	0.900	5 (8.93%)	5 (8.93%)	1.000
Hypertension		204 (57.46%)	70 (66.04%)	0.870	36 (64.29%)	33 (58.93%)	0.560
Diabetes		37 (10.42%)	25 (23.81%)	<0.001	7 (12.50%)	9 (16.07%)	0.590
Congestive heart failure		19 (7.09%)	14 (16.87%)	0.008	5 (8.93%)	7 (12.50%)	0.540
Vascular disease		26 (7.34%)	9 (8.49%)	0.800	3 (5.36%)	2 (3.57%)	0.650
Anticoagulants		185 (51.82%)	68 (64.15%)	0.025	34 (60.71%)	36 (64.29%)	0.700
${\mathrm{CHA}}_{2}$${\mathrm{DS}}_{2}$-Vasc score		2.00 (1.00, 3.00)	3.00 (2.00, 4.00)	<0.001	2.00 (1.00, 3.00)	2.00 (2.00, 3.00)	0.850
Platelet (${\mathrm{10}}^{9}$/L)		178.00 (146.00, 216.00)	177.50 (140.50, 212.00)	0.490	181.00 (143.00, 216.00)	174.50 (137.00, 210.00)	0.510
WBC (${\mathrm{10}}^{9}$/L)		5.56 (4.68, 6.68)	5.92 (4.54, 6.98)	0.260	5.38 (4.63, 6.90)	5.92 (4.70, 7.15)	0.550
Hemoglobin (g/L)		140.00 (128.00, 150.00)	139.00 (127.00, 152.50)	0.810	136.00 (130.00, 153.00)	139.00 (129.00, 153.00)	0.990
NT-proBNP (pg/mL)		302.00 (123.90, 631.20)	1112.00 (677.50, 1922.00)	<0.001	805.70 (344.80, 1474.00)	1125.00 (734.60, 1522.00)	0.150

LAAT, left atrial appendage thrombus; SEC, spontaneous echo contrast; AF, atrial fibrillation; BSA, body surface area; TIA, transient ischemic attack; WBC, white blood cell; NT-proBNP, N-terminal pro b-type natriuretic peptide.

### 3.2 Echocardiographic Characteristics

After PSM, the LAAT/dense SEC group exhibited several notable echocardiographic differences. This group had an increased LA diameter, maximum and minimum LAA areas (LAAmax and LAAmin), but showed reduced LVEF, LAA-EV, LAA-FV, conjunction thickening ratio, aMAPSE, and LAA FAC compared to the non-LAAT/dense SEC group. There were no significant differences in LVEDd, E/e’ ratio, sPAP, LAA ostial diameter, LAA depth, or the number of LAA lobes (Table 2).

**Table 2.** **Echocardiographic parameters LAAT/Dense SEC and Non-LAAT/Dense SEC groups following propensity score matching**.

Variables		Before matching			After matching		
		Non-LAAT/dense SEC (n = 358)	LAAT/dense SEC (n = 107)	*p*	Non-LAAT/dense SEC (n = 56)	LAAT/dense SEC (n = 56)	*p*
Standard echocardiographic parameters							
	LA diameter (mm)	43.00 (40.00, 46.00)	48.00 (44.00, 53.00)	<0.001	45.00 (41.50, 47.50)	48.00 (44.00, 52.00)	<0.001
	LVEDd (mm)	49.00 (46.00, 52.00)	50.00 (47.00, 55.00)	0.003	49.00 (46.00, 52.00)	50.00 (46.00, 56.00)	0.093
	LVEF (%)	61.00 (58.00, 65.00)	57.00 (46.00, 61.00)	<0.001	60.00 (56.00, 63.50)	56.00 (42.00, 61.00)	<0.001
	E/e’	9.06 (7.20, 11.40)	11.40 (8.40, 14.10)	<0.001	10.30 (8.10, 12.25)	11.40 (8.60, 14.10)	0.120
	sPAP (mmHg)	26.00 (22.00, 29.00)	28.00 (24.00, 33.00)	<0.001	26.00 (23.00, 32.00)	27.00 (23.00, 32.00)	0.710
LAA structure parameters							
	Number of LAA lobes	3.15 (1.46)	3.61 (1.35)	0.004	3.40 (1.51)	3.30 (1.27)	0.710
	LAAmax (${\mathrm{cm}}^{2}$)	2.86 (2.22, 3.64)	3.55 (2.66, 4.71)	<0.001	292.72 (221.41, 356.30)	392.37 (275.60, 481.05)	0.001
	LAAmin (${\mathrm{cm}}^{2}$)	0.99 (0.34, 1.98)	2.86 (2.06, 3.70)	<0.001	150.51 (91.39, 245.46)	292.62 (215.59, 371.24)	<0.001
	LAA ostial diameter (mm)	19.00 (16.00, 21.00)	21.00 (19.00, 23.00)	<0.001	19.00 (16.00, 22.00)	21.00 (18.00, 23.00)	0.067
	LAA depth (mm)	26.60 (5.93)	29.59 (6.32)	<0.001	26.98 (6.82)	29.02 (6.68)	0.120
LAA functional parameters							
	LAA-EV (cm/s)	45.00 (32.00, 67.00)	21.50 (17.00, 28.00)	<0.001	32.50 (25.00, 42.00)	22.00 (18.00, 28.00)	<0.001
	LAA-FV (cm/s)	48.00 (34.00, 64.00)	25.00 (20.00, 35.00)	<0.001	39.00 (33.00, 52.00)	25.00 (20.00, 38.00)	<0.001
	Conjunction thickening ratio (%)	22.89 (16.66, 29.19)	11.70 (7.63, 15.51)	<0.001	16.38 (12.14, 21.26)	11.85 (8.93, 14.43)	<0.001
	aMAPSE (mm)	10.83 (8.00, 13.87)	5.09 (4.34, 6.45)	<0.001	8.03 (6.85, 10.25)	5.75 (4.55, 6.59)	<0.001
	LAA FAC (%)	61.86 (26.69)	21.78 (13.23)	<0.001	45.68 (23.97)	22.41 (12.81)	<0.001

LAAT, left atrial appendage thrombus; SEC, spontaneous echo contrast; LA, left atrial; LVEDd, left ventricular end-diastolic diameter; LVEF, left ventricular ejection fraction; sPAP, systolic pulmonary arterial pressure; LAA, left atrial appendage; EV, emptying velocity; FV, filling velocity; LAAmax, maximum left atrial appendage area; LAAmin, minimum left atrial appendage area; aMAPSE, anterior mitral annular plane systolic excursion; FAC, fraction area change.

### 3.3 Correlation of LAAT/Dense SEC with Clinical and Echocardiographic Parameters

**Fig. 3.**
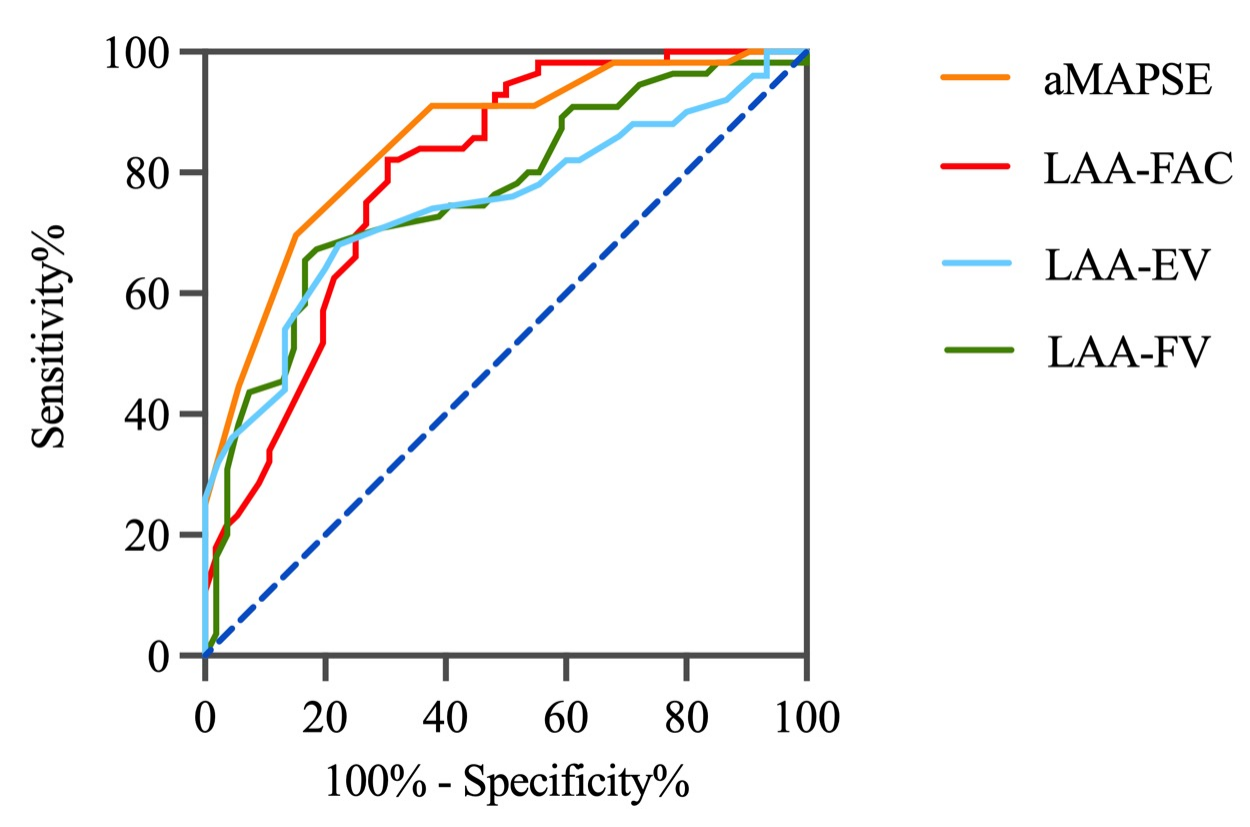
**Diagnostic accuracy of LAA functional parameters.** This figure illustrates the diagnostic performance of various LAA functional parameters in predicting LAAT and SEC. LAA, left atrial appendage; aMAPSE, anterior mitral annular plane systolic excursion; LAAT, left atrial appendage thrombus; SEC, spontaneous echo contrast; FAC, fraction area change; EV, emptying velocity; FV, filling velocity.

Multivariate analysis in the matched cohort revealed that aMAPSE and LAA FAC were independent predictors of LAAT/dense SEC (Table 3). Both LAA FAC and aMAPSE demonstrated a higher diagnostic accuracy in predicting LAAT/dense SEC when compared to LAA-EV and LAA-FV. The diagnostic performance of LAA FAC reached an area under the curve (AUC) of 0.80, with a cutoff value of 29.65%, a sensitivity of 0.70, and a specificity of 0.84. For aMAPSE, the AUC was 0.81, with a cutoff value of 6.76 mm, a sensitivity of 0.81, and a specificity of 0.80 (Fig. 3, Table 4).

**Table 3.** **Univariate and multivariate logistic regression analysis for predicting LAAT/dense SEC in the matched cohort**.

Variables	Univariate analysis		Multivariate analysis	
	OR (95% CI)	*p*	OR (95% CI)	*p*
Gender	1.24 (0.59, 2.61)	0.570		
Age	1.00 (0.95, 1.04)	0.850		
BSA	1.10 (0.14, 8.49)	0.926		
Persistent AF	1.00 (0.30, 3.31)	1.000		
Diabetes	1.34 (0.46, 3.89)	0.590		
Congestive heart failure	1.46 (0.43, 4.90)	0.543		
${\mathrm{CHA}}_{2}$${\mathrm{DS}}_{2}$-VASC	0.94 (0.71, 1.24)	0.670		
Anticoagulants	1.16 (0.54, 2.50)	0.696		
NT-proBNP	1.00 (1.00, 1.00)	0.181		
LA diameter	1.17 (1.07, 1.27)	<0.001		
LVEDd	1.09 (1.02, 1.16)	0.017		
LVEF	0.93 (0.89, 0.97)	0.001		
E/e’	1.09 (0.98, 1.21)	0.103		
sPAP	1.00 (0.96, 1.05)	0.891		
LAA lobes	0.95 (0.72, 1.25)	0.708		
LAAmax	1.00 (1.00, 1.01)	0.002		
LAA ostium	1.08 (0.99, 1.19)	0.093		
LAA depth	1.05 (0.99, 1.11)	0.118		
LAA-EV	0.92 (0.88, 0.96)	<0.001		
LAA-FV	0.94 (0.91, 0.97)	<0.001		
Conjunction thickening ratio	0.90 (0.84, 0.96)	0.001		
aMAPSE	0.47 (0.35, 0.65)	<0.001	0.36 (0.20, 0.66)	0.001
LAA FAC	0.93 (0.91, 0.96)	<0.001	0.94 (0.90, 0.99)	0.023

LAAT, left atrial appendage thrombus; SEC, spontaneous echo contrast; BSA, body surface area; AF, atrial fibrillation; NT-proBNP, N-terminal pro b-type natriuretic peptide; LA, left atrium; LVEDd, left ventricular end-diastolic diameter; LVEF, left ventricular ejection fraction; sPAP, systolic pulmonary arterial pressure; LAA, left atrial appendage; EV, emptying velocity; FV, filling velocity; LAAmax, maximum left atrial appendage area; aMAPSE, anterior mitral annular plane systolic excursion; FAC, fraction area change; OR, odds ratio.

**Table 4.** **Cut-off values and AUC for LAA functional parameters**.

	Cut-off value	Sensitivity	Specificity	AUC	*p*
LAA-EV	33 cm/s	0.80	0.65	0.73	0.046
LAA-FV	30 cm/s	0.83	0.65	0.74	0.046
aMAPSE	6.76 mm	0.81	0.80	0.81	0.037
LAA-FAC	29.65%	0.70	0.84	0.80	0.042

AUC, area under the curve; LAA, left atrial appendage; EV, emptying velocity; FV, filling velocity; aMAPSE, anterior mitral annular plane systolic excursion; FAC, fraction area change.

### 3.4 Correlation of aMAPSE with Clinical and Echocardiographic Parameters

We found the normal aMAPSE group had a lower median age compared to the reduced aMAPSE group. Patients in the reduced aMAPSE group were more likely to have persistent AF, along with higher rates of diabetes and congestive heart failure. Additionally, these patients exhibited significantly elevated ${\mathrm{CHA}}_{2}$${\mathrm{DS}}_{2}$-VASc scores, hemoglobin levels, and N-terminal pro b-type natriuretic peptide (NT-proBNP) levels (Table 5).

**Table 5.** **Demographic and clinical characteristics of patients with normal and reduced aMAPSE**.

Variables		Normal aMPASE (n = 337)	Reduced aMAPSE (n = 128)	*p*
Age (y)		64.50 (57.00, 69.00)	67.00 (62.50, 71.50)	<0.001
Gender				0.180
	Female	132 (39.17%)	59 (46.09%)	
	Male	205 (60.83%)	69 (53.91%)	
BSA ($m^{2}$)		1.87 (0.19)	1.85 (0.18)	0.360
Persistent AF		137 (40.65%)	118 (92.19%)	<0.001
Previous embolic events, TIA, or ischemic stroke		30 (9.01%)	12 (9.45%)	0.880
Hypertension		190 (56.89%)	84 (66.14%)	0.071
Diabetes		36 (10.81%)	26 (20.47%)	0.007
Congestive heart failure		15 (6.00%)	18 (17.82%)	<0.001
Vascular disease		25 (7.49%)	10 (7.94%)	0.820
Anticoagulants		176 (52.38%)	77 (60.63%)	0.110
${\mathrm{CHA}}_{2}$${\mathrm{DS}}_{2}$-Vasc score		2.00 (1.00, 3.00)	3.00 (2.00, 4.00)	<0.001
Platelet (${\mathrm{10}}^{9}$/L)		178.00 (143.00, 217.50)	179.00 (143.00, 209.00)	0.440
WBC (${\mathrm{10}}^{9}$/L)		5.58 (4.68, 6.75)	5.85 (4.60, 6.71)	0.420
Hemoglobin (g/L)		139.00 (127.00, 150.00)	143.00 (130.00, 153.00)	0.023
NT-proBNP (pg/mL)		268.40 (109.20, 593.10)	1012.00 (565.50, 1889.00)	<0.001

aMAPSE, anterior mitral annular plane systolic excursion; AF, atrial fibrillation; BSA, body surface area; TIA, transient ischemic attack; WBC, white blood cell; NT-proBNP, N-terminal pro b-type natriuretic peptide.

### 3.5 Echocardiographic Results in Patients with Normal and Reduced aMAPSE

The study revealed that patients in the reduced aMAPSE group exhibited several significant echocardiographic changes. This group had an increased LA diameter, LVEDd, E/e’ ratio and sPAP, accompanied by a decreased LVEF. Moreover, the reduced aMAPSE group had a larger LAA area with a greater ostial diameter and depth. Furthermore, their LAA-EV, LAA-FV, conjunction thickening ratio and LAA FAC were also reduced. The prevalence of LAAT or dense SEC was also higher in the reduced aMAPSE group (Table 6).

**Table 6.** **Echocardiographic parameters of patients with normal and reduced aMAPSE**.

Variable		Normal aMPASE (n = 337)	Reduced aMAPSE (n = 128)	*p*
Standard echocardiographic parameters				
	LA diameter (mm)	43.00 (40.00, 46.00)	48.00 (44.00, 52.00)	<0.001
	LVEDd (mm)	49.00 (46.00, 52.00)	50.00 (47.00, 55.00)	<0.001
	LVEF (%)	62.00 (58.00, 65.00)	57.00 (47.00, 61.00)	<0.001
	E/e’	8.90 (7.10, 11.40)	11.20 (8.30, 14.00)	<0.001
	sPAP (mmHg)	26.00 (22.00, 29.00)	28.00 (24.00, 32.00)	<0.001
LAA structure parameters				
	Number of LAA lobes	3.22 (1.45)	3.34 (1.42)	0.440
	LAAmax (${\mathrm{cm}}^{2}$)	286.15 (219.49, 366.02)	342.18 (259.01, 438.60)	<0.001
	LAAmin (${\mathrm{cm}}^{2}$)	89.00 (32.78, 190.98)	266.62 (177.78, 333.08)	<0.001
	LAA ostial diameter (mm)	19.00 (16.00, 21.00)	20.00 (18.00, 23.00)	<0.001
	LAA depth (mm)	26.56 (5.74)	29.19 (6.76)	<0.001
LAA functional parameters				
	LAA-EV (cm/s)	47.00 (32.00, 69.00)	23.00 (18.00, 31.00)	<0.001
	LAA-FV (cm/s)	48.00 (34.00, 64.00)	29.00 (20.00, 40.00)	<0.001
	Conjunction thickening ratio (%)	23.73 (17.62, 30.20)	11.37 (7.47, 14.67)	<0.001
	LAA FAC (%)	63.14 (26.87)	24.97 (14.71)	<0.001
	LAAT/dense SEC	20 (5.93%)	87 (67.97%)	<0.001

aMAPSE, anterior mitral annular plane systolic excursion; LA, left atrial; LVEDd, left ventricular end-diastolic diameter; LVEF, left ventricular ejection fraction; sPAP, systolic pulmonary arterial pressure; LAA, left atrial appendage; EV, emptying velocity; FV, filling velocity; LAAmax, maximum left atrial appendage area; LAAmin, minimum left atrial appendage area; FAC, fraction area change; LAAT, left atrial appendage thrombus; SEC, spontaneous echo contrast.

### 3.6 Univariate and Multivariate Analysis of aMAPSE Correlations

Univariate analysis revealed that being male, LVEF, LAA-EV, LAA-FV, conjunction thickening ratio, and LAA FAC were positively correlated with aMAPSE. Conversely, age, persistent AF, diabetes, congestive heart failure, ${\mathrm{CHA}}_{2}$${\mathrm{DS}}_{2}$-VASc score, anticoagulants, NT-proBNP, LA diameter, LVEDd, E/e’ ratio, sPAP, LAAmax, LAAT/dense SEC, LAA ostium, and LAA depth were negatively correlated with aMAPSE. In the multivariate analysis, aMAPSE independently correlated with persistent AF, LAA-EV, conjunction thickening ratio, and LAA FAC (Table 7).

**Table 7.** **Correlations of aMAPSE with clinical and echocardiographic parameters**.

Variables	Univariate analysis		Multivariate analysis	
	OR (95% CI)	*p*	OR (95% CI)	*p*
Gender	0.05 (–0.75, 0.85)	<0.001		
Age	–0.07 (–0.10, –0.03)	0.001		
BSA	1.31 (–0.81, 3.43)	0.224		
Persistent AF	–5.84 (–6.42, –5.27)	<0.001	–1.07 (–2.04, –0.11)	0.030
Diabetes	–1.15 (–2.28, –0.01)	0.049		
Congestive heart failure	–2.09 (–3.55, –0.64)	0.005		
${\mathrm{CHA}}_{2}$${\mathrm{DS}}_{2}$-VASC	–0.32 (–0.58, –0.06)	0.017		
Anticoagulants	–1.69 (–2.47, –0.92)	<0.001		
NT-proBNP	–0.00 (–0.00, –0.00)	<0.001		
LA diameter	–0.30 (–0.36, –0.25)	<0.001		
LVEDd	–0.17 (–0.25, –0.10)	<0.001		
LVEF	0.21 (0.17, 0.25)	<0.001		
E/e’	–0.22 (–0.31, –0.12)	<0.001		
sPAP	–0.14 (–0.20, –0.08)	<0.001		
LAA lobes	–0.24 (–0.52, 0.03)	0.082		
LAAmax	–0.01 (–0.01, 0.01)	<0.001		
LAAT/Dense SEC	–5.71 (–6.47, –4.95)	<0.001		
LAA ostium	–0.25 (–0.35, –0.15)	<0.001		
LAA depth	–0.10 (–0.17, –0.04)	0.001		
LAA-EV	0.11 (0.10, 0.12)	<0.001	0.05 (0.02, 0.07)	<0.001
LAA-FV	0.09 (0.08, 0.11)	<0.001		
Conjunction thickening ratio	0.29 (0.26, 0.32)	<0.001	0.08 (0.04, 0.13)	<0.001
LAA FAC	0.11 (0.10, 0.12)	<0.001	0.04 (0.02, 0.06)	<0.001

aMAPSE, anterior mitral annular plane systolic excursion; BSA, body surface area; AF, atrial fibrillation; NT-proBNP, N-terminal pro b-type natriuretic peptide; LA, left atrium; LVEDd, left ventricular end-diastolic diameter; LVEF, left ventricular ejection fraction; sPAP, systolic pulmonary arterial pressure; LAA, left atrial appendage; LAAmax, maximum left atrial appendage area; LAAT, left atrial appendage thrombus; SEC, spontaneous echo contrast; EV, emptying velocity; FV, filling velocity; FAC, fraction area change.

## 4. Discussion

Assessing LAA function by TEE currently remains complex [8]. This study evaluates the effectiveness of aMAPSE in predicting LAAT/dense SEC in patients with NVAF. The main findings of the current study are: (1) Patients with LAAT/dense SEC exhibited more severely impaired LAA function when compared to those without LAAT or dense SEC, and both aMAPSE, LAA FAC were capable of independently predicting LAAT/dense SEC. (2) Values of aMAPSE <6.76 mm and LAA FAC <29.65% demonstrated higher diagnostic accuracy in predicting LAAT/dense SEC than LAA flow velocity. (3) An independent positive correlation was exhibited between aMAPSE and other LAA functional parameters, such as LAA-EV, conjunction thickening ratio and LAA FAC.

In the current study, patients with increased LA size, impaired LVEF, and deteriorated LAA function were more likely to develop LAAT/dense SEC. This association can be explained by the coupling mechanism between the LA, LV, and LAA. The remolding of the LA in AF is characterized by LA dilation and myocardial fibrosis, which contribute to LA dysfunction and have been linked to LAA SEC and increased risk of thromboembolic events [21, 22, 23]. In AF patients, enlargement of the LA is often accompanied by an increase in left atrial pressure, which raises the LAA afterload, potentially impairing its function and leading to stasis [24, 25]. Furthermore, it’s widely accepted that LVEF is correlated with LAA flow velocity [24, 26]. The LAA undergoes stretching during LV systole and compression during LV diastole, indicating that LAA function is also affected by LV systolic function.

After assessing LAA structure parameters, we demonstrated that NVAF patients with LAAT/dense SEC had a larger LAA area, a finding consistent with previous studies [26, 27, 28, 29, 30]. Additionally, all LAA functional indices were reduced in patients with LAAT/dense SEC compared to those without LAAT/dense SEC. Presently, LAA flow velocity, measured by TEE remains, a key method for assessing LAA function. An LAA-EV of 40 cm/s or less is associated with a higher risk of LAA thrombus formation and subsequent embolic events, while an LAA-EV of 20 cm/s or below often indicates the presence of SEC in the LA/LAA [9]. Recently, LAA strain has emerged as a sensitive marker for assessing LAA function and predicting embolic strokes of undetermined source [31]. However, the practical application is limited since its operation is cumbersome and the requires off-line analysis [31].

Currently, the method of assessing LAA function through TEE is relatively limited and not as well-developed as the evaluation of ventricular and atrial function. The lower surface of the LAA usually overlies the anterior and lateral wall of the LV, and is situated adjacent to the base of the anterior LV wall [14]. Based on this anatomical relationship, we speculate that aMAPSE can be utilized to assess LAA function. This study found aMAPSE correlates well with LAA flow velocity and other LAA function parameters. Additionally, aMAPSE independently correlated with LAAT/dense SEC and aMAPSE <6.76 mm, exhibiting a higher diagnostic accuracy than LAA-EV and LAA-FV in predicting LAAT/dense SEC. These findings suggest that aMAPSE offers incremental value as an LAA functional parameter in the assessment of LAA stasis.

Research by Ono *et al*. [13] demonstrated that LAAEF was an independent determinant of LAAT in NVAF patients with a low CHADS2 score. However, the LAAEF was measured using the Simpson method, which assumes a bullet shaped LAA [13, 32]. This assumption is problematic since the LAA can resemble a finger or stump, and may present in one of four morphological types, with ‘chicken wing’ being the most prevalent (48%), followed by ‘cactus’, ‘windsock’, and ‘cauliflower’ [33]. Therefore, using the Simpson method for calculating LAAEF may not be appropriate. Instead, measuring the LAA FAC using TEE at a suitable angle, may provide a more accurate assessment of LAA function. The current study found that LAA FAC not only correlated independently with LAAT/dense SEC, but accurately predicted LAAT/dense SEC, highlighting its significant potential for assessing the risk of thromboembolic events in NVAF patients.

## 5. Limitations

There are several limitations of this study that warrant consideration. First and foremost, being a single-center study inherently presents potential risks for confounders and biases, which may limit the generalizability of the results. Second, as a cross-sectional investigation, this study did not conclusively establish a correlation between aMAPSE and future thromboembolic events. Finally, the inclusion of only NVAF patients who underwent TEE performed by a single operator limits the study to a specific subset of patients, rather than a more diverse population.

## 6. Conclusions

In conclusion, our findings underscore that both aMAPSE and LAA FAC, characterized by their remarkable accessibility and reproducibility, independently correlated with LAAT or dense SEC and demonstrate high diagnostic accuracy for predicting LAAT or dense SEC. Thus, it may be beneficial to integrate aMAPSE into routine TEE evaluations of LAA function in addition to LAA flow velocity.

## Data Availability

The datasets used and/or analyzed during the current study are available from the corresponding author on reasonable request.
